# The Agglomeration of Manufacturing Industry, Innovation and Haze Pollution in China: Theory and Evidence

**DOI:** 10.3390/ijerph17051670

**Published:** 2020-03-04

**Authors:** Zhidong Liu, Yang Cai, Xiaojing Hao

**Affiliations:** 1 School of Management Science and Engineering, Central University of Finance and Economics, Beijing 100081, China; liuzhidong@cufe.edu.cn; 2 School of Public Finance and Tax, Central University of Finance and Economics, Beijing 100081, China; 2017110010@email.cufe.edu.cn

**Keywords:** haze pollution, industrial agglomeration, innovation, pollution prevention

## Abstract

Haze pollution in China is a serious environmental issue, which does harm both to people’s health and to economic development. Simultaneously, as an important industrial development law, agglomeration may result in the increased concentration of manufacturing firms and, consequently, an increase in haze pollution. However, the positive externalities of agglomeration can also improve the efficiency of regional innovation, which curbs haze pollution. In this paper, we construct both theoretical and empirical models to investigate the effects of industrial manufacturing agglomeration on haze pollution. The results reveal the following: (1) By incorporating the effect of agglomeration and haze pollution into a general endogenous growth model, we show an inverted-U relationship between agglomeration and haze pollution on the balance growth path. (2) Based on data concerning haze pollution (PM_2.5_) and data from 285 Chinese cities, the empirical results verify the findings of the theoretical model. Further, we calculated the values of agglomeration variables, with respect to the inflection points of the inverted-U, which the cities need to reach in order to gain the specific agglomeration values required to enjoy the inhibition effect of agglomeration on haze pollution. (3) A heterogeneity analysis shows that the inverted-U relationship is more obvious among the cities in the middle and northeastern areas of China, as well as medium-size cities. (4) Cities’ environmental regulation policies and high-quality institutional environments can restrain the positive effect of agglomeration on haze pollution. (5) Using three measures of innovation, it is also empirically found that innovation is the mechanism (mediator) between agglomeration and haze pollution.

## 1. Introduction

With the development of urbanization and industrialization, the effect of haze pollution is becoming worse in China. Haze pollution is now more frequent and difficult to control than ever before. According to the China Air Quality Monitoring Platform [1], megalopolises, e.g., the Beijing-Tianjin-Hebei Regions, Yangtze River Delta Region and Pearl River Delta, suffered from haze pollution for over 100 days during 2015. The Chinese government also pays close attention to this problem. For instance, the ‘Plan for the Prevention and Control of Air Pollution’ was issued by the Chinese government in 2013, which set targets and plans to prevent and control the haze pollution. According to the Meteorological Bulletin of the Atmospheric Environment (2018 edition) [2], in China, the average concentration of PM_2.5_ was 39 μg/m^3^ during 2018, which is 9.3% less than that in 2017. The number of hazy days was 20.5 during 2018, which is also 7.1 days less than that in 2017. There is no denying that the effect of the pollution control of the Chinese government is remarkable, however, haze pollution is still an important issue, which concerns the economy and people’s livelihood. A number of researches emphasize the harm of haze pollution to public health, export and economic development [3,4,5]. Markku suggests that about 2.5 million people have died from the effect of air pollution in China [6]. Greenstone and Hanna find that air and water pollution can further affect infant mortality [7].

**Figure 1:**
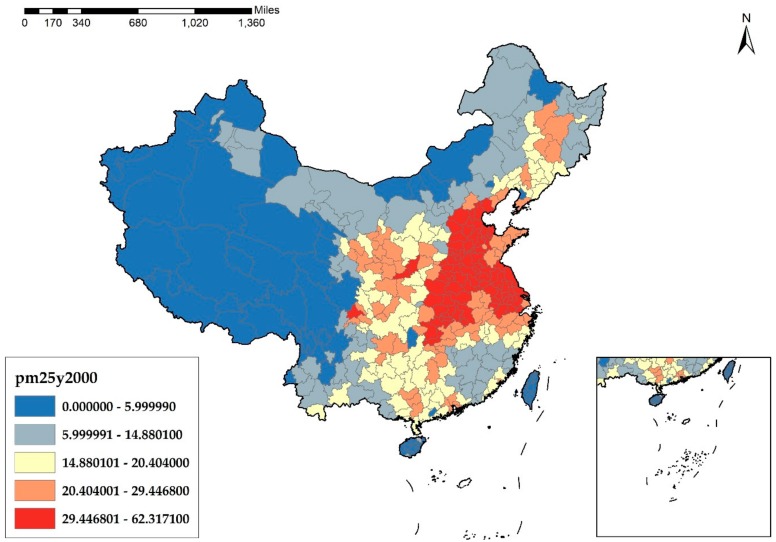
PM_2.5_ Concentration at the city level in China in 2000 (μg/m^3^).

**Figure 2:**
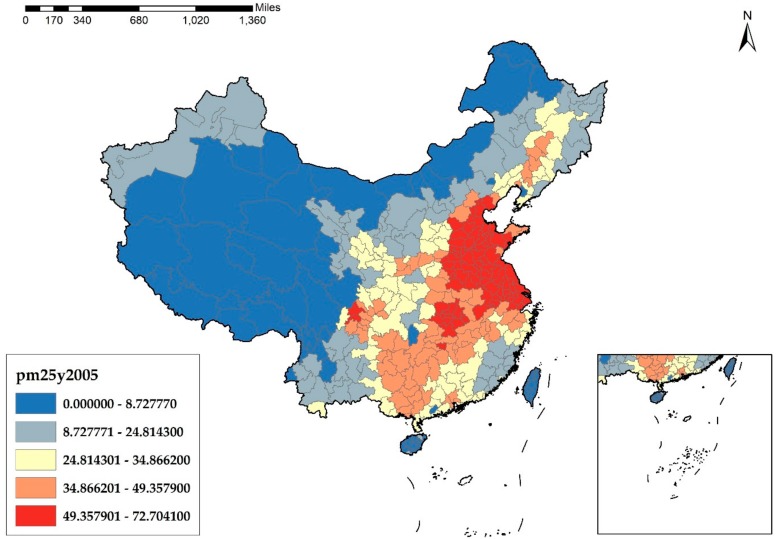
PM_2.5_ Concentration at the city level in China in 2005 (μg/m^3^).

**Figure 3:**
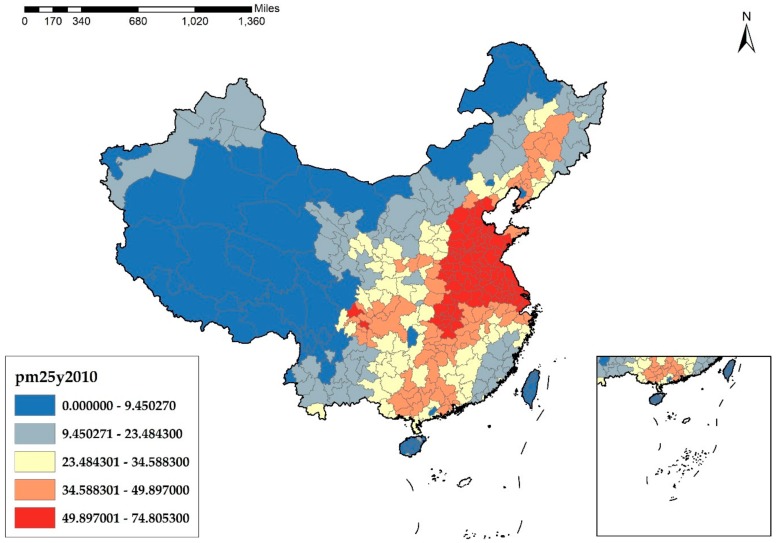
PM_2.5_ Concentration at the city level in China in 2010 (μg/m^3^).

**Figure 4:**
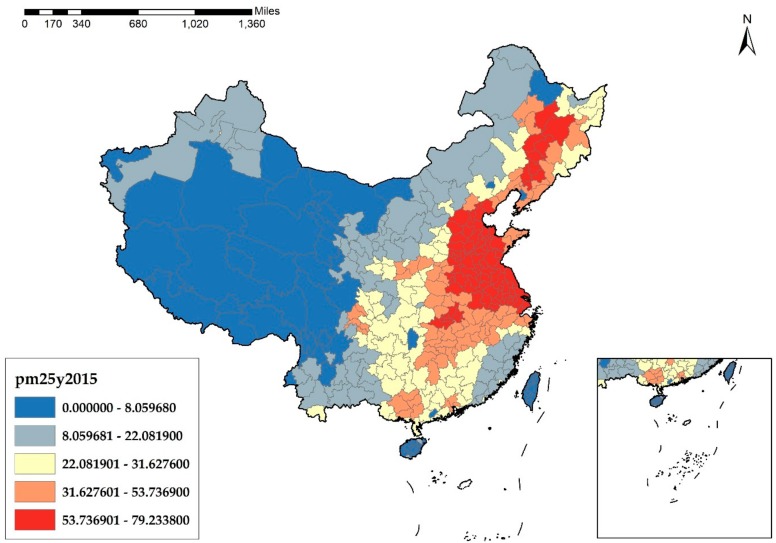
PM_2.5_ Concentration at the city level in China in 2015 (μg/m^3^).

As a widespread environmental problem, understanding the causes of haze pollution is of great importance. In essence, haze pollution derives from the unreasonable structure of energies and industries of China. According to Figure 1, Figure 2, Figure 3 and Figure 4, it is obvious that the cities in the eastern and middle parts of China are suffering more from haze pollution. Xu et al. indicate that more than 70% of emissions, which cause environmental pollution, are generated by the manufacturing industry [8]. Therefore, the large-scale manufacturing industry in the cities of the eastern and middle parts of China is possibly the main cause of the haze pollution. Moreover, in recent years, the spatial distribution of the manufacturing industry has also been showing a clear feature of concentrating in the eastern and middle areas, which results in megalopolises and the agglomeration of the manufacturing industry, and this agglomeration further increases the emissions of pollutants and aggravates the haze problem.

Despite the haze pollution problem, the crowding effect is another potential risk of agglomeration. Agglomeration may lead to a higher land price and more running costs of the infrastructure in a specific area, raising the total costs of the firms in the agglomeration district [9]. Additionally, over-competition also deteriorates the survival environment of firms and aggravates the misallocation of resources [10,11]. Furthermore, agglomeration could result in a crime problem to some degree. Gaigne and Zenou construct an endogenous model, which contains crime and agglomeration, and find that urban agglomeration is positively relative to the per capita crime rate [12].

However, as a general phenomenon during the national economic development, agglomeration also offers some positive externalities. These can be divided into three main types: Marshall, Jacobs and Porter [13,14,15].

- Marshall externalities emphasize the agglomeration of a specific industry and consist of three main effects: intermediate input sharing, labor pooling and knowledge spillover. Firstly, the agglomeration of an industry in a particular area creates a sharing market of the intermediate input, which allows for a better matching between the supply and demand of the production factors. Secondly, a thick labor market is induced by the agglomeration of firms from the same industry. Firms can hire workers with a specific industry skill from this thick labor market to satisfy their production requirement. Thirdly, in the agglomeration district, knowledge spreads between workers and the firm, which facilitates the formation of a local knowledge base. This base induces the generation of new technology and knowledge [16], as well as the transmission of information and skills [13].
- The theory of Jacobs externalities emphasizes the effect of multi-industry agglomeration and also contains the effects of labor pooling, knowledge spillover and public infrastructure sharing in the context of multiple industries [14].
- Porter externalities then illustrate the importance of the competitive edge of industrial agglomeration, whether it is in the context of one or multiple industries [15].

The abovementioned externality theories highlight the positive effect of industrial agglomeration. A number of studies also provide theoretical and empirical support. By inducing agglomeration in a DSGE model, Davis et al. suggested that agglomeration has an economically and statistically significant impact on the growth rate of per capita consumption, raising it by about 10% [17]. Greenstone and Hornbeck compared the firms in and out of the agglomeration district and found that agglomeration increases the total factor productivity of the firms by about 12% [18]. Furthermore, a few studies emphasize the impact of agglomeration on innovation. It was found that agglomeration can raise corporate R&D investment, new product output and innovation efficiency [19,20]. In general, innovation, especially environmental innovation, plays an important role in pollution control. Carrion-Flores and Innes found that environmental innovation (green innovation) is one of the key drivers that led to the decline of toxic emissions in the US [21]. Liu found that technological innovation not only reduces the local haze pollution, but also indirectly leads to the decreasing of the haze pollution of adjacent provinces [22].

According to the above-mentioned analyses, the relation between agglomeration and haze pollution is still ambiguous. On the one hand, industrial agglomeration is actually the spatial aggregation of related firms, which intuitively drives more emissions of pollutants, meaning that agglomeration is one of the important factors that aggravates haze pollution. On the other hand, the positive externalities of agglomeration also motivate innovation. Innovation, especially innovation in environmental technologies, can help to reduce pollution problems, such as haze pollution.

Our work is related to several studies. Conceptually, haze pollution is one kind of environmental pollution, and a number of studies have investigated and discussed the relationship between industrial agglomeration and environmental pollution. For example, based on data from 285 prefecture level cities, Shen et al. [23] adopted the threshold regression, and the results indicated a nonlinear relationship between agglomeration and environmental pollution. Dong et al. [24] utilized a comprehensive index of environmental pollution and found a stably positive relationship between industrial agglomeration and environmental pollution. Zhang et al. [25] also suggested that increased industrial agglomeration has significantly worsened the environmental pollution in China. It is clear that these studies mainly emphasize environmental pollution, and the results and conclusions are controversial. However, there are few studies that effectively and comprehensively discuss the relationship between industrial agglomeration and haze pollution.

A small number of studies focusing on the relationship between agglomeration and haze pollution have been published in recent years. Liu et al. [26] used the dynamic spatial panel model and empirically explored and analyzed the effect of industrial agglomeration on haze pollution by utilizing a panel data of 285 Chinese cities from 2003 to 2012. They found that when other factors are controlled, agglomeration aggravates haze pollution, and this effect varies among different regions of China. Ma et al. [27] empirically examined the spatial pattern and influencing factor of haze pollution within the Yangtze River Delta by applying statistical and spatial econometric models. Additionally, they had similar findings regarding economic agglomeration, i.e., can inhibit haze pollution and has a spatial spillover effect. However, Fan et al. [28] pointed out that the distribution of agglomeration and haze pollution presents a tendency to spread around, and their empirical results show that the agglomeration has a positive spatiotemporal correlation with haze pollution. It is obvious that these studies mainly focus on the net effect of agglomeration on haze pollution; however, their discussion of both the positive and negative impact of agglomeration is insufficient. The study of Xu et al. [8] is relatively more comprehensive and analyzed both of the two sides of the effect of agglomeration on haze pollution, with the empirical finding that the relationship between agglomeration and haze pollution is in an inverted-U shape. However, this work still lacks an effective theoretical analysis and mechanism discussion.

In this study, we explore and discuss how industrial agglomeration affects haze pollution, whether this effect differs according to regional or policy differences, and what the channel between agglomeration and haze pollution is. Compared with related studies, the contributions of this paper are:

- First, as far as we know, there are few or no studies that describe the relationship between agglomeration and haze pollution by employing the endogenous growth framework. Thus, we construct an endogenous growth model to analyze how agglomeration affects haze pollution. Modifying the model of Romer [29], we incorporate a dependence upon agglomeration in innovation. Since it is assumed that economic growth is affected by haze pollution, the above approach makes agglomeration affect both innovation and haze pollution. This endogenous growth model finally predicts an inverted-U relationship between agglomeration and haze pollution.
- Second, we provide empirical evidence to support the findings of the theoretical models. Using city-level data from the Social Economic Data and Application Center of Columbia University and the China Urban Statistical Yearbook from 2003–2016, our panel analysis finds evidence that is consistent with the prediction of the theoretical model. Moreover, this inverted-U relationship is found to be more obvious in the middle region, northeastern region and medium-size cities of China. In addition, cities’ environmental regulation policy and a better institutional environment can reduce the positive effect of agglomeration on haze pollution. Compared with previous studies, our work applies a panel with more observations, and the endogeneity problem of the regression model is a comprehensive issue. In addition, we further employ and analyze the effect of environmental regulation policy and the institutional environment to supplement the existing studies.
- Third, the existing studies generally lack an empirical discussion of the mechanism between agglomeration and haze pollution. Therefore, as a supplement, this study goes a step further and examines that mechanism. Our theoretical analysis suggests that innovation is the mechanism between agglomeration and haze pollution. Therefore, we construct three measures of innovation, including the city-level R&D investment, authorized patents and new product output. Employing mediating effect tests, the effect of innovation as a mediating factor is verified.

In summary, our work provides a richer portrait of how agglomeration affects haze pollution, with theoretical and empirical analysis. The paper is organized as follows: Section 2 describes and deducts the endogenous growth model. Section 3 describes the empirical strategy and data. Section 4 and Section 5 provide and discuss the empirical results. Section 6 concludes the paper, with some discussion of the limitations and recommendations for future research.

## 2. The Theoretical Model

We develop an endogenous growth model that describes the relationship between agglomeration and haze pollution. Our model builds on Romer [29], and the economic system contains the family sector, final consumption goods production sector, intermediate goods production sector and R&D sector. Additionally, there are a few new features in our model: first, agglomeration directly affects the accumulation of innovation, and innovation reduces haze pollution; second, agglomeration is positively relative to economic growth through innovation, and economic growth also aggravates haze pollution. Therefore, the effect of agglomeration on haze pollution depends on both of these two paths.

### 2.1. Preference

Considering a continuous time model, the family sector holds the preference over consumption, leisure and haze pollution:

(1) $$U=\max\int_{0}^{+\infty }e^{-\rho t}\cdot (\ln C_{t}-L_{t}-\gamma \ln H_{t})dt$$

where *C_t_* is the consumption of the unique final goods, and *C_t_*> 0; *L_t_* denotes the labor supply by the family, and *L_t_*> 0; *H_t_* is the haze pollution level (*H_t_*> 0); *γ* indicates the influence coefficient of haze pollution on the total family utility *U*, and *U* > 0; and *ρ* (0 < *ρ <* 1) is the discount factor of the family expected utility. In general, consumption and leisure (more labor supply indicates less leisure) have a positive utility, and haze pollution has a negative utility. Additionally, there exists a budget constraint for the family:

(2) $$s.t.\int_{0}^{+\infty }e^{-Rt}\cdot C_{t}dt=\int_{0}^{+\infty }e^{-Rt}\cdot w\cdot L_{t}dt+K_{0}$$

where $R_{t}\;=\int_{\tau =0}^{t}r_{\tau }\;d\tau$, and this represents the discounted rate or the return on capital in this economic system (*R_t_* > 0); *w* is the wage of labor, and *w* > 0; and *K_0_* is the initial capital owned by the family sector (*K_0_*> 0). By utilizing the budget constraint and optimizing the family utility, we eventually have the dynamic function of family consumption accumulation:

(3) $$\frac{\overset{•}{C}}{C}=r-\rho$$

### 2.2. Production Technology

#### 2.2.1. Final Consumption Goods Production

In this section, the unique final consumption goods *Y* (*Y* > 0) is produced using the labor *L_F_*(*L_F_* > 0) and the intermediate goods *m*(*i*)*,* and *m*(*i*) > 0; and *i* indexes, one kind of unique intermediate good and *i* ∈ [0, *A*], where *A* (*A* > 0) represents the technological level (technology stock) of the whole production sector. We assume that the total labor *L* (*L* > 0) in this economy is constant. Following the specification of Romer, the market of final consumption goods is perfectly competitive, and the price of one unit of a final product equals one [29]. The constant return to scale means that the Cobb–Douglas production function of final goods is

(4) $$Y=L_{F}^{\alpha }\int_{0}^{A}m{\left(i\right)}^{1-\alpha }di$$

where *α* (0 < *α* < 1) indicates the share of the income (output) of labor. During economic operation, the final goods production sector maximizes its profit *π_F_*(*π_F_* > 0):

(5) $$\pi_{F}=\max\left\{L_{F}^{\alpha }\int_{0}^{A}m{\left(i\right)}^{1-\alpha }di-wL_{F}-\int_{0}^{A}p(i)m(i)di\right\}$$

where $L_{F}^{\alpha }\int_{0}^{A}{m(i)}^{1-\alpha }$ denotes the income of the final goods sector (since the price of one unit of a final good equals one); and *p*(*i*) indicates the price of the intermediate goods, and *p*(*i*) > 0. Therefore, *wL_F_* and $\int_{0}^{A}p(i)m(i)\mathrm{di}$ represent the cost of employing labor and purchasing intermediate goods, respectively.

Solving the issue of maximizing the profit (let *π_F_* partially differentiate *m*(*i*) and *L_F_*), we obtain the demand functions of labor and intermediate goods:

(6) $$w=\alpha L_{F}^{\alpha -1}\int_{0}^{A}m{\left(i\right)}^{1-\alpha }di$$

(7) $$p(i)=(1-\alpha )L_{F}^{\alpha }m{\left(i\right)}^{-\alpha }$$

#### 2.2.2. Intermediate Goods Production

The sector of intermediate goods consists of a number of firms, which have monopoly power. These firms borrow capital from the market, and the interest rate equals *r* (*r* > 0; for the lender, it is the return on capital). To produce one unit of an intermediate good, this sector needs to borrow one unit of capital, and therefore, the profit *π_m_* (*π_m_* > 0) of the intermediate goods production sector is

(8) $$\pi_{m}=\max\left\{p(i)m(i)-rm(i)\right\}$$

where *p*(*i*)*m*(*i*) and *rm*(*i*) denote the income and cost of the sector separately. Combining function (7) and (8), we obtain a new expression of *π_m_*, and by solving the issue of maximizing profit, the interest rate of capital *r* can be expressed as:

(9) $$r={\left(1-\alpha \right)}^{2}L_{F}^{\alpha }m{\left(i\right)}^{-\alpha }$$

Therefore, according to function (7)–(9), the maximization of the profit of the intermediate goods production sector equals

(10) $$\pi_{m}{}^{*}=\alpha (1-\alpha )L_{F}^{\alpha }m{\left(i\right)}^{1-\alpha }$$

### 2.3. Innovation Technology

Following the model setup of Romer [29], the innovation $\dot{A}$ in this economic system is produced by the labor *L_I_*(*L_I_* > 0), employed by the R&D sector (*L_I_* can be understood as the labor dedicated to scientific research and innovation), the output efficiency *δ* (*δ* > 0), and the knowledge stock *A*. Additionally, our model employs the effect of agglomeration, which is expressed as *θ* (*θ* > 0). A number of studies have provided theoretical and empirical evidence to support the positive effect of agglomeration on innovation [19,20,30,31]. Therefore, to simplify the model, we assume that $\dot{A}$ is linearly relative to the agglomeration *θ*, innovation output efficiency *δ*, labor *L_I_* and knowledge stock *A*:

(11) $$\overset{•}{A}=\theta \delta L_{I}A$$

In this economic system, the R&D sector needs to borrow money from the financial intermediary to produce innovation, and the financial constraints of the R&D sector depends on the sales of innovation. Thus, the constraint function of the R&D sector is

(12) $$wL_{I}=P_{A}\overset{•}{A}$$

where *wL_I_* is the labor cost of the R&D sector; *P_A_*(*P_A_* > 0) denotes the price of one unit of innovation; hence, $P_{A}\dot{A}$ indicates the value (also sales or income) of the innovation section. Function (12) demonstrates how much labor can be employed under the wage *w*, according to the income from sale of innovation. Further, the intermediate goods production sector buys the innovation from the R&D sector to produce new intermediate goods. According to Romer [29], the price of one unit of innovation equals the discounted value of the profit of the intermediate goods production sector:

(13) $$P_{A}=\int_{0}^{+\infty }\pi_{m}(\tau )\cdot e^{-\int_{t}^{\tau }r(x)dx}d\tau$$

### 2.4. Haze Pollution

Following the setup of Jouvet et al. [32], we assume that haze pollution is positively relative to the total output. To some extent, this assumption is reasonable, according to the environmental Kuznets curve, and similar empirical evidence can be found using our panel. In addition, the relationship between innovation and haze pollution in this economy is set as negative. This setup is in line with the intuition that innovation, especially environmental innovation, can reduce air pollution (e.g., haze pollution). The growth rate is applied in the haze pollution function for these two reasons: first, growth is positively relative to agglomeration, and agglomeration can further result in haze pollution; second, a few studies also offer effective empirical evidence to support this relationship [33,34]. In summary, the haze pollution function is expressed as

(14) $$\overset{•}{H}=g\cdot f(Y,A)$$

where $\dot{H}$ ($\dot{H}$≥0) is the newly formed haze pollution associated with the new emissions of relevant pollutants; and *g* denotes the growth rate of this economic system. To simplify the model, we assume a linear relationship between them:

(15) $$\overset{•}{H}=gY^{\lambda }A^{-\varphi }$$

where *λ* (*λ > 0*) and *φ* (*φ > 0*) are the constraint coefficients and characterize the effect of the output and technology on haze pollution. In order to reach the balance growth path (BGP) technologically, we impose the constraint *λ − φ* = 0.

### 2.5. Equilibrium

Our focus is the equilibrium of the economic system during the BGP. In the equilibrium, the capital market is clearing, and the capital *K* (*K > 0*) offered by the family thus equals the demand of the intermediate goods production sector. Additionally, the demand in the final consumption goods production sector for each intermediate good *i* is the same. Since the market is clearing, the supply of the intermediate goods also equals the demand. Therefore, *m*(*i*) = *m*, where *i*∈[0, *A*], and

(16) $$K=\int_{0}^{A}m(i)di=m\cdot A$$

According to function (16), we can obtain *m = K/A*, and thus the output of the final consumption goods production sector is: ${Y\;=\;(\mathrm{AL}}_{F}{)}^{\alpha }K^{1-\alpha }$. According to Romer [29], the growth rate of *C*, *A*, *Y*, *K* and *H* is the same and equals the growth rate *g* along the BGP. Therefore, based on function (3), the growth rate $g\;=\dot{C}/C=r-\rho$, and *P_A_*, *L_F_*, *L_I_* and *m* are all constant as well. For function (13), by calculating the derivatives of both sides with respect to *t*, we obtain the new expression of the price of innovation:

(17) $$P_{A}=\frac{\pi_{m}}{r}$$

Combining function (3), (6), (10), (12), (16) and (17), we obtain function (18), which demonstrates the relationship between the growth rate *g* and the labor *L_I_* and *L_F_*:

(18) $$\alpha \cdot \frac{L_{I}}{L_{F}}=\frac{\alpha (1-\alpha )}{g+\rho }\cdot g$$

Furthermore, dividing both sides of function (11) by the technology stock *A*, we obtain Equation (19), where (19) is another expression of the growth rate *g*:

(19) $$g=\theta \delta L_{I}$$

In this economic system, as we assumed before, the total labor *L* is constant and consists of *L_F_* and *L_I_*, i.e., *L= L_F_ + L_I_*. Based on this, we can obtain the specific function of the growth rate *g* when combing function (18) and (19):

(20) $$g=\frac{(1-\alpha )\theta \delta L-\rho }{2-\alpha }$$

Further, by calculating the partial derivatives of the growth rate *g* to agglomeration *θ*, we obtain the derivative of *g*:

(21) $$\frac{\partial g}{\partial \theta }=\frac{(1-\alpha )\delta L}{2-\alpha }>0$$

According to function (21) and the value range of each parameter, the value of this partial derivative is always greater than zero, which indicates that agglomeration and economic growth are positively related. Our panel also supports this finding, and the theoretical mechanism in this model is: agglomeration promotes the development of innovation, and innovation increases economic growth. Moreover, the interest rate *r* can be further expressed as

(22) $$r=\rho +g={\left(1-\alpha \right)}^{2}L_{F}^{\alpha }{\left(\frac{K}{A}\right)}^{-\alpha }$$

Based on (22), *K/A* equals

(23) $$\frac{K}{A}={\left[\frac{{\left(1-\alpha \right)}^{2}}{\rho +g}\right]}^{\frac{1}{\alpha }}L_{F}$$

According to the output of this economy (*Y*) and the original function of haze pollution ($\dot{H}$), we obtain (*λ* – *φ* = 0)

(24) $$\overset{•}{H}=gL_{F}^{\lambda \alpha }{\left(\frac{K}{A}\right)}^{\lambda (1-\alpha )}$$

Combining function (23) and (24), we can obtain the expression of $\dot{H}$, whose parameters are all constant and greater than zero:

(25) $$\overset{•}{H}=g{\left(L-\frac{g}{\theta \delta }\right)}^{\lambda }{\left[\frac{{\left(1-\alpha \right)}^{2}}{\rho +g}\right]}^{\frac{1-\alpha }{\alpha }\lambda }$$

Based on the chain rule of derivation, we calculate the derivative of haze pollution $\dot{H}$ with respect to agglomeration *θ*:

(26) $$\begin{array}{r} \frac{d\overset{•}{H}}{d\theta }=\frac{d\overset{•}{H}}{dg}\cdot \frac{dg}{d\theta }=\frac{(1-\alpha )\delta L}{2-\alpha }\cdot {\left(L-\frac{g}{\theta \delta }\right)}^{\lambda -1}\cdot {\left[\frac{{\left(1-\alpha \right)}^{2}}{\rho +g}\right]}^{\frac{1-\alpha }{\alpha }\lambda -1}\cdot \frac{{\left(1-\alpha \right)}^{2}}{\theta \delta {\left(\rho +g\right)}^{2}}\times \\ \left[\frac{(1-2\alpha )\lambda }{\alpha }g^{2}+(\theta \delta L-\rho -\rho \lambda -\frac{1-\alpha }{\alpha }\lambda \theta \delta L)g+\rho \theta \delta L\right] \end{array}$$

Function (26) shows that haze pollution is affected by agglomeration. To analyze how agglomeration influences haze pollution more intuitively, we extract the positive common factor, which is $\frac{(1-\alpha )\delta L}{2-\alpha }\cdot {\left(L-\frac{g}{\theta \delta }\right)}^{\lambda -1}\cdot {\left[\frac{{\left(1-\alpha \right)}^{2}}{\rho +g}\right]}^{\frac{1-\alpha }{\alpha }\lambda -1}\cdot \frac{{\left(1-\alpha \right)}^{2}}{\theta \delta {\left(\rho +g\right)}^{2}}$, and then it can be understood that the effect of agglomeration on haze pollution mainly depends upon function (27):

(27) $$\frac{(1-2\alpha )\lambda }{\alpha }g^{2}+(\theta \delta L-\rho -\rho \lambda -\frac{1-\alpha }{\alpha }\lambda \theta \delta L)g+\rho \theta \delta L$$

Function (27) is a quadratic function. In general, the Cobb–Douglas function *α* represents the share of the income of labor, and a number of studies suggest that it is between 60% and 70% in China (e.g., Fève et al. set *α = 2/3* [35]). Thus, the coefficient of the square term (*1-2α*)*λ/α < 0*, which means function (27), is in an inverted-U shape. If *g = 0*, function (27) equals *ρθδL*, which is greater than zero, indicating that the intercept of this quadratic function is positive. Moreover, we calculate the value of *g* at the inflection point of the function:

(28) $$\frac{\left(1-\alpha \right)\lambda \theta \delta L-\alpha (\theta \delta L-\rho -\rho \lambda )}{2(1-2\alpha )\lambda }$$

**Figure 5:**
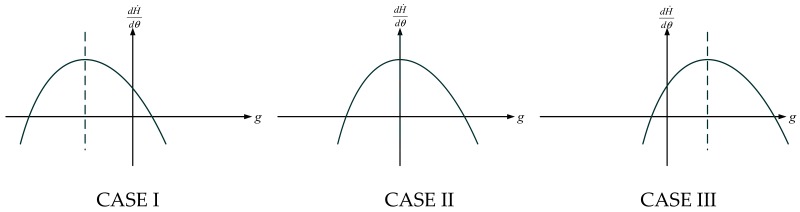
Three cases according to function (29).

According to function (28), the images of function (27) are divided into three cases (Figure 5, the ordinate and abscissa denote $d\dot{H}/d\theta$ and *g*, respectively), with respect to the following three conditions:

(29) $$\left\{\begin{array}{l} \frac{\left(1-\alpha \right)\lambda \theta \delta L-\alpha (\theta \delta L-\rho -\rho \lambda )}{2(1-2\alpha )\lambda }<0,\;\mathrm{CASE}\;I\\ \frac{\left(1-\alpha \right)\lambda \theta \delta L-\alpha (\theta \delta L-\rho -\rho \lambda )}{2(1-2\alpha )\lambda }=0,\;\mathrm{CASE}\;\mathrm{II}\\ \frac{\left(1-\alpha \right)\lambda \theta \delta L-\alpha (\theta \delta L-\rho -\rho \lambda )}{2(1-2\alpha )\lambda }>0,\;\mathrm{CASE}\;\mathrm{III} \end{array}\right.$$

All these cases demonstrate that there is only one positive zero point of (27), indicating that if *g* > 0, the effect of agglomeration on haze pollution is always positive before the certain positive zero point and negative after it. More specifically, the relationship between agglomeration and haze pollution is in an inverted-U shape, and the corresponding mechanisms are: first, agglomeration stimulates innovation, and innovation can reduce the haze pollution of this economy; second, through innovation, agglomeration also promotes economic growth, and economic growth causes more pollutant emissions and aggravates the haze pollution problem. Thus, there exists a balance between the abovementioned two powers, and finally, the relationship is shown to be an inverted-U. This inverted-U relationship is also found in some related studies that discuss carbon emissions, eco-efficiency and growth [36,37].

## 3. Model Specification, Variables and Data

### 3.1. Basic Model Specification

Ehrlich and Holdren [38] propose the IPAT model and emphasize that, in terms of the environment, the pressure is generated from three factors, i.e., *I = P × A × T*, where *I*, *P*, *A* and *T* represent the environmental pressure, population, economic development and technology level, respectively. The IPAT model has been widely used in related studies. However, the linear equivalence between the variables is not consistent with reality. Therefore, Dietz and Rosa [39] created the STIRPAT model, which modifies the IPAT and further incorporates the random item to facilitate an empirical analysis. The basic STIRPAT model is: $I_{\mathrm{it}}{=\alpha \times P}_{\mathrm{it}}^{\theta }{\times A}_{\mathrm{it}}^{\gamma }{\times T}_{\mathrm{it}}^{\varphi }{\times \varepsilon }_{\mathrm{it}}$, where *i* is the region; *t* denotes the year; *α* represents the constant item; and *θ*, *γ* and *φ* indicate the estimated coefficient of population, economic development and technology, respectively. Based on the STIRPAT model, we further introduce agglomeration (*agg*) into the model, and haze pollution (HP) is regarded as environmental pressure. Since the nonlinear relationship between agglomeration and haze pollution is shown in our theoretical model, we introduce the square term of the agglomeration variable as well. Therefore, the basic empirical model is as follows:

(30) $$HP_{it}=\beta_{0}+\beta_{1}agg_{it}+\beta_{2}agg\_sq_{it}+\sum \beta X+\sum F+\varepsilon_{it}$$

where *agg* represents the degree of agglomeration; *agg_sq* denotes the square of the agglomeration variable; *β_0_* is the constant; *X* is the vector of the control variables; *F* is the vector of the relevant fixed effect (we control both the city fixed effects and year fixed effects); *ε* is the random error term; and *i* and *t* denote the city and year, respectively.

### 3.2. Variable Description

#### 3.2.1. Explained Variable

Haze pollution (HP). PM_2.5_ now is the primary pollutant in China, according to the Ministry of Environmental Protection of China. Thus, we employ the average concentration of PM_2.5_ in each city as the proxy of regional haze pollution. However, there are few official data on the PM_2.5_ concentration of each city before 2013. Therefore, we collect the data from the Socioeconomic Data and Applications Center and use the ArcGIS to extract the concentration of PM_2.5_ in the cities that are at the prefecture level or above and calculate the average value of the PM_2.5_ in each year at the city level. These data are consistent with the information reported by the Ministry of Environmental Protection.

#### 3.2.2. Key Explanatory Variable

Agglomeration (agg_empl, agg_output). Since the manufacturing industry accounts for about 70% of pollutant emissions, this paper uses the agglomeration of the manufacturing industry as the key explanatory variable. Due to the fact that the agglomeration variable illustrates the degree of the agglomeration level of one specific industry, we use the location quotient to measure it, which is expressed as:

(31) $$agg_{jit}=\frac{e_{jit}/e_{it}}{E_{jct}/E_{ct}}$$

where *e_jit_* is the size of industry *j* in city *i* in year *t*; *e_it_* denotes the size of all industries in city *i* in year *t*; *E_jit_* is the national size of industry *j* in year *t*; and *E_it_* denotes the total size of all industries at the national level. To ensure the robustness of the results, we measure two aspects of agglomeration: agg_empl and agg_output, which represent the agglomeration of employment and industrial output, respectively, and a larger location quotient indicates a higher degree of industrial agglomeration.

#### 3.2.3. Control Variables

Economic development (agdp). The basic STIRPAT model shows that economic growth is one of the important factors affecting emissions, and our theoretical model also employs this setup. In the empirical analysis, following related studies [26,40], we use the logarithm of the per capita GDP to measure the economic development.

Population (popu_den). An increment in the population may result in more demand of consumption goods and, in turn, may therefore lead to more emission of pollutants. To measure and control the effect of population, we use the population density, which is calculated as: the total population divided by the urban area.

Industrial structure (seco_indu, tert_indu). Different industrial structures lead to different energy consumption structures. In general, the energy consumption of the secondary industry is remarkably higher than that of other industries, indicating that its pollutant emissions are higher than those of other industries as well. On the contrary, a relatively larger scale of tertiary industry indicates not only less energy consumption, but also a potentially better innovation system, which can be instrumental in the reduction of pollutant emissions. Thus, the proportion of the added value of secondary and tertiary industries are employed as the control variables.

Technological level (tech). The technological process promotes the adoption of cleaner production by firms and therefore reduces the emission of pollutants and mitigates the haze problem. We use the proportion of scientific and technological fiscal expenditure in the total fiscal expenditure to measure the urban technological level. This measure indicates both the aspiration of the government to innovate and, as a few studies show, the significant effect of fiscal expenditure on innovation and growth [41,42].

Financial development level (fin_dev). A developed financial market makes financial support more accessible to firms, which drives economic growth and facilitates corporate innovation. According to previous analysis, innovation is theoretically relative to pollutant emissions. Thus, we further control this, and we use the per capita deposit to measure the financial development level.

Foreign direct investment (fdi). Generally, foreign enterprises are more likely to transfer their polluting capacity to a host in other countries due to the weaker environmental regulation of the local government, which leads to serious pollution problems. However, foreign firms can also introduce cleaner production technologies into the firms and industries of host countries through technology transformation and the spillover effect, which has the opposite effect of contributing to the reduction of pollutant emissions [43]. Thus, the logarithm of the actual use of FDI is used to control the effect of foreign direct investment.

Infrastructure (infra). The construction of urban infrastructure affects the ability of the talent attractiveness and development potential of the city, indicating that infrastructure could possibly affect urban growth and innovative vitality. Therefore, the infrastructure may also influence haze pollution. From a more comprehensive perspective, we apply the logarithm of the number of Internet users to measure the infrastructure.

Greening level (green). Green plants mitigate haze pollution effectively in cities. Thus, on the basis of the important role of green plants in curbing haze pollution, we employ the urban greening rate to measure the greening level of each city.

### 3.3. Data and Summary Statistics

To empirically analyze how agglomeration influences haze pollution, we collected data from 285 cities at the prefecture level or above in China from 2003 to 2016. The explained variable, haze pollution, is described by the PM_2.5_ concentration. These data are derived from the Socioeconomic Data and Applications Center of Columbia University, which measures the PM_2.5_ concentration through satellite monitoring. The data on the key explanatory variable and the control variables are from the China Statistical Yearbook, China City Statistical Yearbook, China Statistical Yearbook for the Regional Economy. Table 1 shows the summary statistics of the main variables in our analysis.

## 4. Empirical Results

### 4.1. Baseline Regression

Table 2 reports the results of the baseline findings. In order to ensure the robustness of our results, we used two agglomeration measures, agg_empl and agg_output. In models (1) and (2), the control variables are not included in the regression, and the coefficient of the square term of agg_empl is significantly negative at the 5% level, while the coefficient of agg_output_sq is not statistically significant, indicating that an inverted-U relationship between agglomeration and haze pollution possibly exists. Models (3) and (4) contain control variables. The coefficients of agg_empl and agg_output are both positive and statistically significant, while the agg_empl_sq and agg_output_sq’s coefficients are significantly negative, illustrating that the effect of agglomeration on haze pollution is in an inverted-U shape, i.e., with the improvement of agglomeration, haze pollution first increases; and when the agglomeration reaches and exceeds a certain degree, haze pollution starts to decline. These findings support the results of our theoretical model. According to the results of model (3) and (4), we also calculate the location of the inflection point of the inverted-U. According to models (3) and (4), the values of the location quotient with respect to the infection point are 1.3258 (agg_empl) and 5.7227 (agg_output), indicating that in order to enjoy the inhibition effect of agglomeration on haze pollution, the city must reach and pass this specific degree of agglomeration, i.e., agg_empl ≥ 1.3258 and agg_output ≥ 5.7227.

### 4.2. Instrumental Variable Regression

Empirical analysis generally needs to solve endogenous problems. The reasons for endogenous problems include omitted variables, sample selection bias, reverse causality, etc. First, our model controls the factors that can affect haze pollution as far as possible; however, there may still exist some variables, which are not well considered, and the omitted variable problem could bias the estimated results. Second, as for the sample selection bias, we basically employ all the cities of China at prefecture level or above. Cities that are not included in our analysis may affect the results as well. Third, according to our theoretical model, we mainly analyze how agglomeration influences haze pollution, while haze pollution also has an impact on the entrance of firms, since severe haze pollution can become the driving force of the environmental regulation of the local government. This regulation, to some extent, prevents the entry of firms with a pollution potential, e.g., the entry of manufacturing firms, and therefore impacts agglomeration, which means that the reverse causality problem in our empirical model cannot be neglected.

To solve the endogenous problem, in this part we exploit the instrumental variable method to re-estimate the results. Finding effective instrumental variables is of great importance, and a valid instrumental variable contains the following characteristics: first, relevance: the instrumental variable should have a strong correlation with the explanatory variable; second, exogeneity: the instrumental variable should be exogenous, i.e., the instrumental variable should be independent of unmeasured confounding; and third, exclusion: exclusion suggests that the instrumental variable affects the explained variable only through the explanatory variables. Therefore, we use the explanatory variable with a three-year lag (agg_empl_it-3_, agg_output_it-3_) and the dummy variable, i.e., whether the city had owned a railway in 1933 (rail_1933), to construct the instrumental variable group.

The first instrumental variable (agg_empl_it-3_, agg_output_it-3_) follows the setup and suggestions of a number of previous studies [44,45], and its validity reflects whether the historical data of the explanatory variable can affect its present state; however, the present will not influence the past. In order to ensure the exogeneity of this instrumental variable, we exploited the explanatory variable with a three-year lag as the first instrumental variable. The second variable (rail_1933) also follows the rules of an effective instrumental variable. The dummy variable, i.e., whether the city owned a railway in 1933, is exogenous from the perspective of time; furthermore, some studies suggest that the railway is an important incentive to generate industrial agglomeration [46,47]. In addition, there are no other effective paths through which this dummy variable can affect haze pollution. In summary, this instrumental variable satisfies the characteristics of relevance, exogeneity and exclusion.

To empirically examine the significance of our instrumental variable group, we employed a series of tests. Models (5) and (6) in Table 2 show the results. The Kleibergen–Paap rk LM statistics of models (5) and (6) are 214.261 and 79.240, respectively, and the corresponding *p*-value is close to zero, indicating that there exists no under-identification problem of the instrumental variable group. The Kleibergen–Paap rk Wald F statistics of these two models are 689.271 and 54.958, respectively, and are both greater than the value of the 10% level of Stock–Yogo statistics, suggesting that there is no weak instrumental variable problem, i.e., these instrumental variables are strongly relative to the explanatory variable. Moreover, the Hansen J statistics of both models are 0.735 and 0.559, and the corresponding *p*-values are both greater than 0.1, indicating that our instrumental variable group satisfies the exogenous rules. Based on our instrumental variable group, the results of the 2SLS estimation show that the coefficients of agg_empl and agg_output are still significantly positive, and the coefficients of agg_empl_sq and agg_output_sq are significantly negative, illustrating that the inverted-U shape relationship between agglomeration and haze pollution is robust after solving the endogenous problem. In the 2SLS regression, the city fixed effects are not included, since the instrumental variable rail_1933 is a dummy variable at city level.

### 4.3. Summary of Robustness Check

We employ various robustness checks in this part. For our main explained variable, PM_2.5_ (HP), we alternatively use the logarithm of the PM_2.5_ concentration (lnHP), and the results are shown in models (1) and (2) of Table 3. The coefficients of agg_empl_sq and agg_empl_sq are still negative and statistically significant at the 5% significance level, illustrating that the inverted-U relationship still exists when we substitute the explained variable.

We also consider the possible omitted variable. During production, the explained variable PM_2.5_ is closely related to the emission of SO_2_, while the SO_2_ emission, as well as the PM_2.5_, can affect innovation, agglomeration and growth, which leads to an omitted variable bias in our model. Thus, models (3) and (4) further control the effect of SO_2_ using the SO_2_ emission per unit area (so2_den). The results indicate that the SO_2_ emission is positively relative to the PM_2.5_ emission, and the estimated coefficients of the agglomeration variables are similar.

To further eliminate the endogenous problem, we lag the explanatory variables and all of the control variables with a one-year lag. Models (5) and (6) suggest that the inverted-U relationship is still significant. We also consider the effect of a few special cities, i.e., cities that are under the direct control of the state council in China. Therefore, the samples of these four cities are dropped in the regression of models (7) and (8). Since Guangzhou has similar characteristics to the above four cities, its estimation is also dropped. The results of models (7) and (8) demonstrate that our previous findings are robust.

We also consider the range of the value of the explained variable. Since the PM_2.5_ concentration is greater than zero, we exploit the Tobit model to re-estimate the results by setting the lower limit as zero. The results of models (9) and (10) are similar and indicate the robustness of previous empirical findings.

In reality, we control the city fixed effect in the regression, so that the impact of neglected relative factors is reduced to some extent. However, to further guarantee the robustness of the estimation, more related environmental variables are also considered during the estimation. We include the variables of the urban area (area), average humidity (humi), average city altitude (atli), average surface water resources (sur_wat) and whether the city is a coastal city (cos_city). We collect these data from the China Environmental Yearbook, the annual data set of the National Meteorological Information Center and Baidu Map. The results (Table A1, Table A2 and Table A3) are shown in the Appendix A and are similar to our previous findings as well.

### 4.4. Heterogeneous Analysis

#### 4.4.1. Location

In this part, we examine how the city heterogeneity affects the relationship between agglomeration and haze pollution. A typical reality of China is the unbalanced development between different regions. In general, the cities in the eastern regions are in the possession of greater industrial agglomeration and are also affected more by the haze pollution problem. Therefore, in the analysis, we need to consider the location differences of cities. The traditional classification method of the Chinese regions cannot satisfy the present needs of economic development. This paper follows the ‘Division method of the east, west, middle and northeast regions’, published by the National Bureau of Statistics of China, and divides the full sample into four subsamples: eastern, middle, western and northeastern region cities. The regression results of the classified samples are displayed in Table 4.

It is shown that the coefficients of the square term of the agglomeration variables are all negative; however, only in the samples of the middle and northeastern region cities are the coefficients negative and statistically significant. This indicates that the inverted-U relationship between agglomeration and haze pollution is more obvious in the middle and northeastern cities. A possible reason is that the developments of the eastern and western region cities are significantly higher or lower than the average level, respectively. Thus, the corresponding effects of agglomeration may be far beyond or beneath the inflection point of the inverted-U, and this inverted-U relationship is therefore not so evident in these subsamples. On the contrary, the developments of cities in the middle and northeastern regions are around average, and the agglomeration levels are just close to the inflection point of the inverted-U. Consequently, this inverted-U relationship is more obvious among the cities of these two regions. Besides, in model (3), only the coefficient of agg_empl is significantly positive, indicating that the positive effect of agglomeration on haze pollution is still greater than its negative effect in the middle region to some extent.

#### 4.4.2. City Size

The impact of agglomeration on haze pollution also varies according to city size. Cities with a greater size generally have a greater energy consumption and relevant industries, i.e., larger cities are more likely to have a greater industrial agglomeration level and more pollutant emissions. However, the positive externalities and accumulation of technologies or the human capital of larger-size cities also benefit the reduction of pollution. According to the ‘Adjustment of the Standards for the Classification of Urban Size’, published by the State Council of China, the cities are divided into five kinds. To simplify our analysis, we reclassify the cities into three categories: the cities whose population is less than half million are defined as small cities; cities whose population is between half a million and one million are defined as medium cities; and cities whose population is greater than one million are all defined as large cities. Table 5 reports the regression results of the above three subsamples.

The coefficients of agg_empl and agg_empl_sq in model (3) are significantly positive and negative, respectively, which is consistent with our previous analysis and indicates the inverted-U relationship between agglomeration and haze pollution among medium-size cities. However, this inverted-U relationship is not evident among large and small cities. The possible reason for the results is similar to the explanation of 4.4.1: the agglomeration levels of large- or small-size cities are far higher or lower than the level of the inflection point. Thus, this inverted-U characteristic is not obvious among these subsamples. This explanation is reasonable, because the cities in the eastern or western regions are always larger or smaller than the average city size, and differences in the industrial agglomeration level therefore lead to these results.

## 5. Further Discussion

### 5.1. Environmental Regulation Policy

The environmental regulation policies of the government always play an important role in pollution control. The State Council of China published the ‘Plan of Acid Rain Control and SO_2_ Pollution Regulation Areas’ in 1998, dividing the cities into environmentally regulated and unregulated areas and setting a series of environmental standards in the regulated area, such as the average concentration of SO_2_ and the pH value of the rainwater. Therefore, we employ this policy as a moderator and estimate its effect in our model. First, according to the plan, we generate the dummy variable envi_regu, which represents whether this city is in a regulated area (envi_regu=1 if the city is in a regulated area) and then include the interaction term of envi_regu and the agglomeration variables in the regression. We report the regression results in Table 6.

The coefficients of envi_regu are all negative and statistically significant at 1% in models (1)– (3), which indicates that this policy significantly reduces the haze pollution of the cities in environmentally regulated areas. Furthermore, the coefficients of the interaction terms are significantly negative, suggesting that environmental regulation weakens the positive effect of agglomeration on haze pollution. According to the environmental regulation policy, these cities are required to limit their pollutant emissions and also rehabilitate existing high-polluting firms. The entrance of new firms is also affected by the policy, e.g., high-polluting enterprises are prohibited to enter the market of these cities, and only firms with better environmental technology are welcomed and share the positive externalities of industrial agglomeration. Therefore, this environmental regulation policy can effectively reduce the augmenting effect of industrial agglomeration on haze pollution.

### 5.2. The Quality of the Institutional Environment

We also considered the moderating effect of the institutional environment in our analysis. Some studies have emphasized the importance of institutional quality, e.g., Zakaria and Bibi find that in South Asia, a 1% improvement in institutional quality can decrease pollution by about 0.114% [48], and Huynh and Hoang suggest that FDI initially increases the air pollution in Asia. However, the improvement of institutional quality can help to decrease this effect beyond a certain threshold [49]. Hence, this part further analyzes the moderating effect of institutional quality. We generate the institutional quality variable following Wang et al., which is the Marketization Index of each province in China [50]. This index has six aspects: the relationship between the government and market, the development of the non-state-owned economy, the development of the product market, the development of the factor market, and the development of the market intermediary organization and legal environment, including 23 basic indicators. Since the Marketization Index measured by Wang et al. covers a wide range of dimensions and is vertically and horizontally comparative, it is proper to employ it as a proxy that estimates the regional quality of the institutional environment. According to the Marketization Index, we generate a dummy variable high_inst to represent the quality of the institutional environment of each region. If the Marketization Index value of a specific region is greater than the median value of all of the regions’ value in year t, then high_inst=1. Then, in the regressions, we generate the interaction term of high_inst and agglomeration terms. Table 7 reports the results.

Models (1)–(3) show that the coefficients of high_inst are all negative and statistically significant at the 1% level, illustrating that a higher quality institutional environment can help to decrease haze pollution, which is consistent with related studies as well. Moreover, the coefficients of the interaction terms are also significantly negative in models (2) and (3), which further verifies the moderating effect of the institutional environment quality and indicates that a better institutional environment quality can further weaken the positive effect of industrial agglomeration on haze pollution. Possible explanations for this result are as follows. First, cities with a better institutional environment quality generally pay more attention to environmental protection, since they need to maintain their political status and attract development resources, such as high-quality firms. Second, a better institutional environment quality benefits corporate innovation [51]. With the development of technological innovation, especially green innovation, the haze pollution is alleviated in these regions. Both paths effectively restrain the positive effect of industrial agglomeration on haze pollution.

### 5.3. Mechanism Analysis

In our theoretical model, the mediator between agglomeration and haze pollution is innovation. Agglomeration first promotes the production of knowledge and contributes to economic growth. Economic growth then leads to the haze pollution problem. Innovation further helps to curb haze pollution, and consequently, there exists a balance between industrial agglomeration and haze pollution. Our theoretical model indicates an inverted-U shape of this relationship, and the above empirical analysis provides evidence of these findings using city-level data from China. In this part, we test the mechanism of innovation in our panel.

There are various measures of innovation. In order to ensure the robustness of our results, we follow some related studies [52,53] and construct three measures of innovation. From the perspective of the life cycle of innovation, first, we use the logarithm of the total R&D investment at the city level to measure innovation (innovation_rd). We calculate this indicator by summing up the R&D investment of the firms in one specific city. The data are derived from the China Annual Survey of Industrial Enterprises from 2003 to 2013. A number of industrial variables in the China Statistical Yearbook are calculated on the basis of this database, which guarantees the validity of our measures. Second, we also employ the logarithm of the output of a new product at the city level as another innovation measure (innovation_np), and the estimation method is similar to R&D investment. Third, to capture the intermediate product of innovation, we further exploit the logarithm of one plus the number of urban patent authorization (innovation_p), and the data are collected from the CNKI patent database manually. Eventually, we have three innovation measures, R&D investment, patent authorization and the output of a new product.

To estimate whether innovation is the mediator, we use the mediating effect test. We construct the following identification strategy:

(32) $$HP_{it}=\beta_{0}+\beta_{1}agg_{it}+\beta_{2}agg\_sq_{it}+\sum \beta X+\sum F+\varepsilon_{it}$$

(33) $$Innovation_{it+1}=\beta_{0}+\beta_{1}agg_{it}+\sum \beta X+\sum F+\varepsilon_{it}$$

(34) $$HP_{it}=\beta_{0}+\beta_{1}agg_{it}+\beta_{2}agg\_sq_{it}+\beta_{1}innovation_{it-2}+\sum \beta X+\sum F+\varepsilon_{it}$$

where innovation is denoted by the aforementioned three innovation measures. If agglomeration is significantly positively related to innovation, and the absolute value of the coefficient or the significance of agg_sq decreases when bringing the innovation variable into regression, then the mediating effect of innovation is acknowledged. In (32), we do not employ the square term of agglomeration for these two reasons: first, in our theoretical model, we assume that agglomeration is positively related to innovation; second, the results show that both the coefficients of agglomeration variables and its square term are not significant when the model contains them. However, when (32) only includes the variable of agglomeration, its coefficient is significantly positive, which supports the assumption in our theoretical model as well. Moreover, in (32) and (33), we also consider the lag effect of technology generation and transformation. Table 8 reports the results of the mediating effect tests of innovation.

In models (2) and (5) in Panel A, B and C, the coefficients of agg_empl and agg_output are mainly positive and statistically significant, illustrating that agglomeration is positively related to the innovation. This result is consistent with the prediction of our theoretical model and also provides empirical evidence of a potential mechanism between agglomeration and haze pollution. According to models (3) and (6) of these panels, the coefficients of the innovation variables are significantly negative, which indicates a reduction in the impact on haze pollution caused by innovation, and the rationality of our theoretical model is tested as well. Furthermore, both the absolute value of the coefficients of agg_empl, agg_empl_sq, agg_output, and agg_output_sq in these models are decreased, and their significance levels are also decreased. These results confirm the mediating effect of innovation between agglomeration and innovation. Overall, the evidence in Section 4 and Section 5 identifies an inverted-U relationship between agglomeration and haze pollution, and the effect of city heterogeneity, environmental regulation, institutional quality, and the mechanism behind this inverted-U.

## 6. Conclusions

In this paper, we study how industrial agglomeration affects haze pollution by constructing an endogenous growth model and conducting an empirical analysis. According to our theoretical model, industrial agglomeration first promotes innovation through its positive externalities and then increases economic growth. Economic growth further raises the emissions of pollutants and causes haze pollution, while innovation helps to curb it. We find that during the balanced growth path of this economy, there is a balance between agglomeration and haze pollution, and the effect of industrial agglomeration on haze pollution follows an inverted-U shape, i.e., agglomeration first increases haze pollution, before reaching a specific degree of agglomeration; then, when agglomeration passes this point, its effect becomes negative. Using data from 285 cities in China, this inverted-U relationship is empirically supported. We also calculate the values of agglomeration with respect to the inflection point of the inverted-U. For the location quotient of employment, this specific degree is 1.3258, while for the output, it is 5.7227. Furthermore, this inverted-U is more obvious among the middle and northeastern region cities and medium-size cities; also, an environmental regulation policy and better institutional environment quality can weaken the positive effect of industrial agglomeration on haze pollution. Finally, in a mechanism analysis, we empirically examine the mediating effect of innovation and further confirm the role of innovation as the path between agglomeration and haze pollution.

Our work is related to studies that focus on how industrial agglomeration affects haze pollution and provides a reference for the construction of a theoretical and empirical model on this issue. Furthermore, we discuss the mechanisms between agglomeration and haze pollution, which extends and also offers a reference for the research on the mediating variables pertaining to this issue.

However, there still exist some limitations of this study. First, in the theoretical analysis section, we employ an endogenous growth model to describe the relationship between agglomeration and haze pollution. This model simplifies the economic system to some extent, which is not only an advantage, but also a disadvantage. A simplified model helps us to emphasize the research issue; however, the impact of other factors of the economic system may be neglected. Second, in the empirical analysis section, while we use several measures to improve our identification strategy as far as possible (e.g., the instrumental variable method), there may still exist some factors that cause biased estimation, such as the endogeneity problem, the measures of variables, and the variables’ spatial correlation problem. Third, for our research issue, we mainly discuss industrial agglomeration and haze pollution, and innovation is considered as the channel between them. However, there are some other kinds of agglomeration (e.g., financial agglomeration), and new mechanisms between industrial agglomeration and haze pollution, which can be explored.

Therefore, for future studies, these improvements and research directions are recommended: a more reasonable theoretical and empirical model construction, an analysis of the impact of different kinds of agglomeration, and discussion of the mechanisms from multiple perspectives.

In general, haze pollution is still an important environmental issue in China at present. In view of above findings and conclusions, the policy suggestions of this paper are:

(1) The government needs to dialectically consider the relationship between industrial agglomeration and haze pollution. Even now, a number of cities are still facing a haze pollution problem. However, in the long term, pushing industrial agglomeration may benefit economic growth and reduce haze pollution. For the cities with lower agglomeration levels, the local government should encourage and introduce new firms to form greater industrial agglomeration and generate a positive effect of agglomeration. With respect to the cities with higher agglomeration levels, they need to exert their own radiation effect and industrial advantages to drive the surrounding areas to form their own industrial agglomeration advantages.

(2) The implementation of industrial and environmental regulation policies needs to pay attention to the heterogeneities of cities. Generally, the cities in the eastern area are more developed, and the effect of the positive externalities of agglomeration is going further. For these cities, they should better exert the advantages of agglomeration and flexibly use environmental regulation policies. However, for the cities in the western area, development should be the primary goal, and environmental regulation policy needs to better coordinate with industrial policy, so that these regions can take the road toward economic and industrial development with local characteristics.

(3) The government should strengthen the role of innovation in the process of economic development and pollution regulation. As the key path between industrial agglomeration and haze pollution in this paper, innovation is now playing a more important role in the economy and pollution regulation. Thus, the government needs to transform the existing development modes and promote innovation-driven strategies to eventually realize green growth.

## Figures and Tables

**Table 1:** Summary statistics.

Variables	Observations	Mean	Std. Dev	Min	Median	Max
HP	3920	36.4603	16.3482	4.5171	34.1226	90.8564
agg_empl	3976	0.8681	0.4723	0.0211	0.7866	2.8415
agg_output	3974	0.7450	0.6986	0.0000	0.5458	12.0520
agdp	3968	10.0748	0.8218	4.5951	10.1133	13.0557
popu_den	3976	5.7230	0.9085	1.5475	5.8638	7.8817
seco_indu,	3990	0.4851	0.1144	0.0000	0.4899	0.9097
tert_indu	3990	0.3662	0.0903	0.0000	0.3584	0.8023
tech	3976	0.0094	0.0119	0.0000	0.0055	0.2068
fin_dev	3976	9.7254	1.1054	7.1556	9.6144	13.7257
fdi	3972	0.0218	0.0236	0.0000	0.0140	0.3405
infra	3953	12.4144	1.2436	5.4661	12.4292	17.7617
green	3974	0.3648	0.1465	0.0000	0.3821	3.8664

**Table 2:** Basic regression and IV regression.

Variables	(1)	(2)	(3)	(4)	(5)	(6)
	HP	HP	HP	HP	HP-2SLS	HP-2SLS
agg_empl	1.8121		3.2138 ***		9.1861 ***	
	(1.1485)		(1.1480)		(1.8106)	
agg_empl_sq	−0.9401 **		−1.2120 ***		−5.8078 ***	
	(0.4384)		(0.4489)		(0.8069)	
agg_output		−0.0343		0.9225 **		5.7692 ***
		(0.3765)		(0.3978)		(1.6503)
agg_output_sq		−0.0001		−0.0806 **		−1.9811 ***
		(0.0300)		(0.0328)		(0.4963)
Kleibergen–Paap rk LM					214.261	79.240
(*p*-value)					(0.0000)	(0.0000)
Kleibergen–Paap rk Wald F					689.271	54.958
Stock–Yogo 10%					13.43	13.43
Hansen J					0.735	0.559
(*p*-value)					(0.3912)	(0.4546)
Control vars	NO	NO	YES	YES	YES	YES
Observations	3918	3916	3888	3888	3059	3057
R-squared	0.9469	0.9469	0.9481	0.9481	0.5491	0.4900
City fixed	YES	YES	YES	YES	NO	NO
Year fixed	YES	YES	YES	YES	YES	YES

Note: Robust standard errors are in parentheses. ***, ** indicates 1% and 5% significance level, respectively. Control vars indicates whether the regression employs control variables. City fixed and Year Fixed indicates whether in the empirical model the city fixed effect and year fixed effect are controlled.

**Table 3:** Robustness check.

Variables	(1)	(2)	(3)	(4)	(5)
	ln HP	ln HP	HP	HP	HP
agg_empl	0.1015 ***		2.9685 ***		
	(0.0358)		(1.1374)		
agg_empl_sq	−0.0340 **		−1.0926 **		
	(0.0140)		(0.4407)		
agg_output		0.0258 **		0.8390 **	
		(0.0102)		(0.3975)	
agg_output_sq		−0.0023 **		−0.0752 **	
		(0.0009)		(0.0326)	
so2_den			0.0720 ***(0.0224)	0.0730 ***(0.0228)	
					
L.agg_empl					0.8982
					(1.2553)
L.agg_empl_sq					−0.6376
					(0.4870)
Control vars	YES	YES	YES	YES	L.YES
Observations	3888	3888	3888	3888	3609
R-squared	0.9588	0.9587	0.9588	0.9587	0.9495
City fixed	YES	YES	YES	YES	YES
Year fixed	YES	YES	YES	YES	YES
**Variables**	**(6)**	**(7)**	**(8)**	**(9)**	**(10)**
	**HP**	**HP**	**HP**	**HP**	**HP**
agg_empl		3.2212 ***		3.2138 ***	
		(1.1535)		(1.1023)	
agg_empl_sq		−1.2185 ***		−1.2120 ***	
		(0.4515)		(0.4311)	
agg_output			0.9426 **		0.9225 **
			(0.4005)		(0.3820)
agg_output_sq			−0.0818 **		−0.0806 **
			(0.0329)		(0.0315)
L.agg_output	0.7587 *				
	(0.4281)				
L.agg_output_sq	−0.0772 **				
	(0.0330)				
Control vars	L.YES	YES	YES	YES	YES
Observations	3609	3819	3819	3888	3888
R-squared	0.9495	0.9479	0.9478		
Pseudo R-Square				0.3511	0.3510
City fixed	YES	YES	YES	YES	YES
Year fixed	YES	YES	YES	YES	YES

Note: Robust standard errors are in parentheses. ***, ** indicates 1% and 5% significance level, respectively. Control vars indicates whether the regression employs control variables. City fixed and Year Fixed indicates whether in the empirical model the city fixed effect and year fixed effect are controlled.

**Table 4:** Heterogeneous analysis—location.

Variables	(1)	(2)	(3)	(4)	(5)	(6)	(7)	(8)
	Eastern Regions		Middle Regions		Western Regions		Northeastern Regions	
	HP	HP	HP	HP	HP	HP	HP	HP
agg_empl	2.4584		4.3982 *		1.3316		7.1170 **	
	(1.8319)		(2.5338)		(1.6950)		(3.5150)	
agg_empl_sq	−1.0592		−1.3170		−0.4239		−3.4423 *	
	(0.6733)		(1.2576)		(0.7248)		(1.9409)	
agg_output		1.5870		4.0602 ***		0.7959		5.9417 **
		(1.0352)		(1.1703)		(0.6458)		(2.8241)
agg_output_sq		−0.3512		−0.9740 **		−0.0580		−2.8889 **
		(0.3069)		(0.3851)		(0.0485)		(1.1832)
Control vars	YES	YES	YES	YES	YES	YES	YES	YES
Observations	1186	1186	1105	1105	1291	1291	459	459
R-squared	0.9653	0.9652	0.9309	0.9313	0.9308	0.9308	0.9395	0.9397
City fixed	YES	YES	YES	YES	YES	YES	YES	YES
Year fixed	YES	YES	YES	YES	YES	YES	YES	YES

Note: Robust standard errors are in parentheses. ***, **, * indicates 1%, 5% and 10% significance level, respectively. Control vars indicates whether the regression employs control variables. City fixed and Year Fixed indicates whether in the empirical model the city fixed effect and year fixed effect are controlled.

**Table 5:** Heterogeneous analysis—city size.

Variables	(1)	(2)	(3)	(4)	(5)	(6)
	Large Cites		Medium Cities		Small Cities	
	HP	HP	HP	HP	HP	HP
agg_empl	0.3970		6.5832 ***		4.8768 *	
	(1.7488)		(2.1724)		(2.5224)	
agg_empl_sq	−0.3874		−2.4613 **		−1.3768	
	(0.6352)		(0.9771)		(1.0018)	
agg_output		1.4339		0.8469		1.7376
		(1.0474)		(0.6975)		(1.4887)
agg_output_sq		−0.1614		−0.0688		−0.5055
		(0.2990)		(0.0525)		(0.3094)
Control vars	YES	YES	YES	YES	YES	YES
Observations	2003	2003	1236	1236	649	649
R-squared	0.9376	0.9377	0.9485	0.9480	0.9380	0.9374
City fixed	YES	YES	YES	YES	YES	YES
Year fixed	YES	YES	YES	YES	YES	YES

Note: Robust standard errors are in parentheses. ***, **, * indicates 1%, 5% and 10% significance level, respectively. Control vars indicates whether the regression employs control variables. City fixed and Year Fixed indicates whether in the empirical model the city fixed effect and year fixed effect are controlled.

**Table 6:** Moderating effect—environmental regulation.

Variables	(1)	(2)	(3)
	HP	HP	HP
envi_regu	−3.6095 ***	−5.5417 ***	−3.4595 ***
	(0.4051)	(0.6195)	(0.4391)
agg_empl		1.5957 *	
		(0.9005)	
agg_empl_sq		−2.3072 ***	
		(0.4278)	
agg_empl×envi_regu		−4.5955 ***	
		(0.6795)	
agg_output			−1.2515 **
			(0.5051)
agg_output_sq			−0.2178 ***
			(0.0517)
agg_output×envi_regu			−2.5933 ***
			(0.4940)
Control vars	YES	YES	YES
Observations	3888	3888	3888
R-squared	0.5444	0.8135	0.8123
City fixed	NO	NO	NO
Year fixed	YES	YES	YES

Note: Robust standard errors are in parentheses. ***, **, * indicates 1%, 5% and 10% significance level, respectively. Control vars indicates whether the regression employs control variables. City fixed and Year Fixed indicates whether in the empirical model the city fixed effect and year fixed effect are controlled.

**Table 7:** Moderating effect—institutional environment.

Variables	(1)	(2)	(3)
	HP	HP	HP
high_inst	1.6073 ***	4.2673 ***	2.7425 ***
	(0.3049)	(0.6178)	(0.4278)
agg_empl		3.7406 ***	
		(1.1416)	
agg_empl_sq		−0.6210	
		(0.4475)	
agg_empl×high_inst		−3.1661 ***	
		(0.6165)	
agg_output			2.5260 ***
			(0.5821)
agg_output_sq			−0.1923 ***
			(0.0457)
agg_output×high_inst			−1.9235 ***
			(0.4701)
Control vars	YES	YES	YES
Observations	3888	3888	3888
R-squared	0.9441	0.9446	0.9444
City fixed	YES	YES	YES
Year fixed	YES	YES	YES

Note: Robust standard errors are in parentheses. *** indicates 1% significance level, respectively. Control vars indicates whether the regression employs control variables. City fixed and Year Fixed indicates whether in the empirical model the city fixed effect and year fixed effect are controlled.

**Table 8:** Mechanism analysis.

**Panel A. R&D Investment**						
**Variables**	**(1)**	**(2)**	**(3)**	**(4)**	**(5)**	**(6)**
	**HP**	**F.innovation-rd**	**HP**	**HP**	**F.innovation-rd**	**HP**
agg_empl	3.2138 ***	0.3421 ***	1.9656 *			
	(1.1480)	(0.1103)	(1.1921)			
agg_empl_sq	−1.2120 ***		−0.8476 *			
	(0.4489)		(0.4554)			
L2.innovation_rd			−0.1220 **			−0.1174 **
			(0.0523)			(0.0521)
agg_output				0.9225 **	0.0781	−0.0402
				(0.3978)	(0.0553)	(0.4424)
agg_output_sq				−0.0806 **		−0.0021
				(0.0328)		(0.0350)
Control vars	YES	YES	YES	YES	YES	YES
Observations	3888	3661	3337	3888	3661	3337
R-squared	0.9481	0.9651	0.9542	0.9481	0.9650	0.9541
City fixed	YES	YES	YES	YES	YES	YES
Year fixed	YES	YES	YES	YES	YES	YES
**Panel B. Patent Authorization**						
**Variables**	**(1)**	**(2)**	**(3)**	**(4)**	**(5)**	**(6)**
	**HP**	**F.innovation-p**	**HP**	**HP**	**F.innovation-p**	**HP**
agg_empl	3.2138 ***	0.2350 ***	2.2013 *			
	(1.1480)	(0.0576)	(1.1832)			
agg_empl	−1.2120 ***		−0.9849 **			
	(0.4489)		(0.4551)			
L2.innovation_p			−0.7009 ***			−0.6764 ***
			(0.1508)			(0.1502)
agg_output				0.9225 **	0.1398 *	−0.0132
				(0.3978)	(0.0721)	(0.4375)
agg_output_sq				−0.0806 **		−0.0061
				(0.0328)		(0.0351)
Control vars	YES	YES	YES	YES	YES	YES
Observations	3888	3661	3337	3888	3661	3337
R-squared	0.9481	0.9523	0.9544	0.9481	0.9524	0.9544
City fixed	YES	YES	YES	YES	YES	YES
Year fixed	YES	YES	YES	YES	YES	YES
**Panel C. Output of New Product**						
**Variables**	**(1)**	**(2)**	**(3)**	**(4)**	**(5)**	**(6)**
	**HP**	**F.innovation-np**	**HP**	**HP**	**F.innovation-np**	**HP**
agg_empl	3.2138 ***	0.0126	1.8106			
	(1.1480)	(0.0466)	(1.1870)			
agg_empl_sq	−1.2120 ***		−0.8189 *			
	(0.4489)		(0.4533)			
L2.innovation_np			−0.0434			−0.0409
			(0.0763)			(0.0763)
agg_output				0.9225 **	0.0553 **	0.6871
				(0.3978)	(0.0269)	(0.4290)
agg_output_sq				−0.0806 **		−0.0580 *
				(0.0328)		(0.0339)
Control vars	YES	YES	YES	YES	YES	YES
Observations	3888	3661	3337	3888	3661	3612
R-squared	0.9481	0.9235	0.9541	0.9481	0.9235	0.9492
City fixed	YES	YES	YES	YES	YES	YES
Year fixed	YES	YES	YES	YES	YES	YES

Note: Robust standard errors are in parentheses. ***, **, * indicates 1%, 5% and 10% significance level, respectively. Control vars indicates whether the regression employs control variables. City fixed and Year Fixed indicates whether in the empirical model the city fixed effect and year fixed effect are controlled.

## Appendix A

**Table A1:** Control for area and altitude.

Variables	(1)	(2)	(3)	(4)
	HP	HP	pm25	pm25
agg_empl	6.5557 ***		7.4042 ***	
	(1.2905)		(1.3286)	
agg_empl_sq	−3.8684 ***		−4.6680 ***	
	(0.5685)		(0.5808)	
agg_output		2.3968 ***		1.0579 *
		(0.6000)		(0.6228)
agg_output_sq		−0.3750 ***		−0.2733 **
		(0.1130)		(0.1192)
area	6.3233 ***	7.3120 ***		
	(0.5227)	(0.5419)		
alti			−0.0062 ***	−0.0063 ***
			(0.0004)	(0.0004)
Control vars	YES	YES	YES	YES
Observations	3888	3888	3754	3754
R-squared	0.5695	0.5649	0.5706	0.5596
City fixed	NO	NO	NO	NO
Year fixed	YES	YES	YES	YES

Note: Robust standard errors are in parentheses. ***, **, * indicates 1%, 5% and 10% significance level, respectively. Control vars indicates whether the regression employs control variables. We only control the year fixed effect, since according to the data source, the added control variables are not changed by year for each city.

**Table A2:** Control for humidity and surface water resource.

Variables	(1)	(2)	(3)	(4)
	HP	HP	HP	HP
agg_empl	9.8708 ***		7.5707 ***	
	(1.3584)		(1.2541)	
agg_empl_sq	−5.1154 ***		−4.1684 ***	
	(0.5920)		(0.5459)	
agg_output		2.3602 ***		0.9823 *
		(0.6826)		(0.5639)
agg_output_sq		−0.4339 ***		−0.2099 *
		(0.1549)		(0.1079)
humi	−0.3538 ***	−0.3742 ***		
	(0.0245)	(0.0239)		
sur_wat			−4.1810 ***	−4.3001 ***
			(0.1886)	(0.1881)
Control vars	YES	YES	YES	YES
Observations	3754	3754	3860	3860
R-squared	0.5774	0.5698	0.6331	0.6263
City fixed	NO	NO	NO	NO
Year fixed	YES	YES	YES	YES

Note: Robust standard errors are in parentheses. ***, **, * indicates 1%, 5% and 10% significance level, respectively. Control vars indicates whether the regression employs control variables. We only control the year fixed effect, since according to the data source, the added control variables are not changed by year for each city.

**Table A3:** Control for costal and comprehensive control.

Variables	(1)	(2)	(3)	(4)
	HP	HP	HP	HP
agg_empl	4.9142 ***		2.5170 **	
	(1.2311)		(1.1147)	
agg_empl_sq	−2.8131 ***		−1.0168 **	
	(0.5393)		(0.4670)	
agg_output		2.8757 ***		2.7573 ***
		(0.6314)		(0.4593)
agg_output_sq		−0.4483 ***		−0.2386 ***
		(0.1341)		(0.0452)
cos_city	−11.1289 ***	−12.1684 ***	−9.5015 ***	−10.0769 ***
	(0.7468)	(0.7352)	(0.6298)	(0.6234)
lnarea			4.9531 ***	5.5201 ***
			(0.4488)	(0.4597)
alti			−0.0081 ***	−0.0082 ***
			(0.0004)	(0.0004)
humi			0.0338	0.0151
			(0.0307)	(0.0303)
lnsur_wat			−4.1545 ***	−4.0673 ***
			(0.2377)	(0.2363)
Control vars	YES	YES	YES	YES
Observations	3888	3888	3726	3726
R-squared	0.5967	0.5966	0.7053	0.7083
City fixed	NO	NO	NO	NO
Year fixed	YES	YES	YES	YES

Note: Robust standard errors are in parentheses. ***, **, * indicates 1%, 5% and 10% significance level, respectively. Control vars indicates whether the regression employs control variables. We only control the year fixed effect, since according to the data source, the added control variables are not changed by year for each city.

## References

[1] China Air Quality Monitoring Platform. https://www.aqistudy.cn/.

[2] Meteorological Bulletin of the Atmospheric Environment (2018 Edition). http://www.nmc.cn/publish/environment/National-Bulletin-atmospheric-environment.htm.

[3] Grossman G.M., Krueger A.B. (1995). Economic growth and the environment. Q. J. Econ..

[4] Ebenstein A., Fan M., Greenstone M., He G., Yin P., Zhou M. (2015). Growth, pollution, and life expectancy: China from 1991–2012. Am. Econ. Rev..

[5] Hering L., Poncet S. (2014). Environmental policy and exports: Evidence from Chinese cities. J. Environ. Econ. Manag..

[6] Markku K. (2015). China’s choking cocktail. Nature.

[7] Greenstone M., Hanna R. (2014). Environmental regulations, air and water pollution, and infant mortality in India. Am. Econ. Rev..

[8] Xu C., Lou X.H., Qin M. (2019). The impact of geographical agglomeration and urban size on haze pollution: An empirical study based the data of Chinese cities. J. Chongqing Univ. (Soc. Sci. Ed.).

[9] Fujita M., Thisse J.F. (2013). Economics of Agglomeration: Cities, Industrial Location, and Globalization.

[10] Fontagne L., Santoni G. (2019). Agglomeration economies and firm-level labor misallocation. J. Econ. Geogr..

[11] Kaya A., Koc M. (2019). Over-Agglomeration and its effects on sustainable development: A case study on Istanbul. Sustainability.

[12] Gaigné C., Zenou Y. (2015). Agglomeration, city size and crime. Eur. Econ. Rev..

[13] Marshall A. (1890). Principles of Economics.

[14] Jacobs J. (1969). The Economy of Cities.

[15] Porter M. (1998). Clusters and the new economics of competition. Harv. Bus. Rev..

[16] Jaffe A., Trajtenberg M., Henderson R. (1993). Geographic localization of knowledge spillovers as evidenced by patent citations. Q. J. Econ..

[17] Morris A., Davis J.D., Fisher M., Toni M.W. (2014). Macroeconomic implication of agglomeration. Econometrica.

[18] Greenstone M., Hornbeck R. (2010). Identifying agglomeration spillovers: Evidence from winners and losers of large plant openings. J. Political Econ..

[19] Capello R. (1999). Spatial transfer of knowledge in high technology milieus: Learning versus collective learning processes. Reg. Stud..

[20] Martin P., Mayer T., Mayneris F. (2008). Spatial concentration and plant-level productivity in France. J. Urban Econ..

[21] Carmen E.C.F., Robert I. (2010). Environmental innovation and environmental performance. J. Environ. Econ. Manag..

[22] Liu X.H. (2018). Dynamic evolution, spatial spillover effect of technological innovation and haze pollution in China. Energy Environ..

[23] Shen N., Zhao Y., Wang Q. (2018). Diversified agglomeration, specialized agglomeration, and emission reduction effect—A nonlinear test based on Chinese city data. Sustainability.

[24] Dong F., Wang Y., Zheng L., Li J.Y., Xie S.X. (2020). Can industrial agglomeration promote pollution agglomeration? Evidence from China. J. Clean. Prod..

[25] Zhang K.K., Xu D.Y., Li S.R. (2019). The impact of environmental regulation on environmental pollution in China: An empirical study based on the synergistic effect of industrial agglomeration. Envrion. Sci. Pollut. Res..

[26] Liu J., Zhao Y.H., Cheng Z.H., Zhang H.M. (2018). The effect of manufacturing agglomeration on haze pollution in china. Int. J. Environ. Res. Public Health.

[27] Ma R.F., Wang C.C., Jin Y.X., Zhou X.J. (2019). Estimating the effects of economic agglomeration on haze pollution in Yangtze River Delta China using an econometric analysis. Sustainability.

[28] Fan Q., Yang S., Liu S. (2019). Asymmetrically spatial effects of urban scale and agglomeration on haze pollution in China. Int. J. Environ. Res. Public Health.

[29] Romer P. (1990). Endogenous technological change. J. Political Econ..

[30] Katz M., Shapiro C. (1986). Technology adoption in the presence of network externalities. J. Political Econ..

[31] Li Y., Ran X.J., Wu Y.Y. (2017). A study on the impact of logistics industry agglomeration on regional innovation. Agro Food Ind. Hi Tech.

[32] Jouvet P., Pestieau P., Ponthiere G. (2010). Longevity and environmental quality in an OLG model. J. Econ..

[33] Fei N., Liu H.Y. (2015). Correlation analysis of FDI, environmental pollution and economic growth: An empirical examination based on dynamic simultaneous equation model. J. Int. Trade.

[34] Greiner A. (2011). Environmental pollution, the public sector and economic growth: A comparison of different scenarios. Optim. Control Appl. Methods.

[35] Fève P., Moura A., Pierrard O. (2019). Shadow banking and financial regulation: A small-scale DSGE perspective. J. Econ. Dyn. Control.

[36] Jalil A., Feridun M. (2011). The impact of growth, energy and financial development on the environment in China: A cointegration analysis. Energy Econ..

[37] Bai Y., Deng X.Z., John G., Zhao Z., Xu H. (2019). How does urbanization affect residential CO_2_ emissions? An analysis on urban agglomerations of China. J. Clean. Prod..

[38] Ehrlich P.R., Holdren J.P. (1971). Impacts of population growth. Science.

[39] Dietz T., Rosa E.A. (1994). Rethinking the environmental impacts of population, affluence and technology. Hum. Ecol. Rev..

[40] Wang Y., Han R., Jumpei K. (2016). Is there an environmental kuznets curve for SO_2_ emissions? A semi-parametric panel data analysis for China. Renew. Sustain. Energy Rev..

[41] Lin B.Q., Zhu J. (2019). Fiscal spending and green economic growth: Evidence from China. Energy Econ..

[42] Ling C., Yang W.H. (2019). R&D tax credits and firm innovation: Evidence from China. Technol. Forecast. Soc. Change.

[43] Asghari M. (2013). Does FDI promotes MENA region’s environment quality? Pollution halo or pollution haven hypothesis. Int. J. Sci. Res. Environ. Sci..

[44] Baiocchi M., Cheng J., Small D.S. (2014). Instrumental variable methods for causal inference. Stat. Med..

[45] Sovey A.J., Green D.P. (2011). Instrumental variables estimation in political science: A readers’ guide. Am. J. Political Sci..

[46] Huang T., Xi J.C., Ge Q.S. (2019). Spatial differentiation and integration optimization of an urban agglomeration tourism system under the influence of high-speed railway network evolution. Appl. Spat. Anal. Policy.

[47] Hodgson C. (2018). The effect of transport infrastructure on the location of economic activity: Railroads and post offices in the American West. J. Urban Econ..

[48] Zakaria M., Bibi S. (2019). Financial development and environment in South Asia: The role of institutional quality. Environ. Sci. Pollut. Res..

[49] Huynh C.M., Hoang H.H. (2019). Foreign direct investment and air pollution in Asian countries: Does institutional quality matter?. Appl. Econ. Lett..

[50] Wang X.L., Fan G., Yu J. (2017). China Provincial Marketization Index Report (2016).

[51] Zhou Y.X. (2014). Role of institutional quality in determining the R&D investment of Chinese firms. China World Econ..

[52] Amore M., Schneider C., Zaldokas A. (2013). Credit supply and corporate innovation. J. Financ. Econ..

[53] Chemmanur T.J., Loutskina E., Tian X. (2014). Corporate venture capital, value creation, and innovation. Rev. Financ. Stud..

